# Circulating miR-21, miR-29a, and miR-126 are associated with premature death risk due to cancer and cardiovascular disease: the JACC Study

**DOI:** 10.1038/s41598-021-84707-7

**Published:** 2021-03-05

**Authors:** Hiroya Yamada, Koji Suzuki, Ryosuke Fujii, Miyuki Kawado, Shuji Hashimoto, Yoshiyuki Watanabe, Hiroyasu Iso, Yoshihisa Fujino, Kenji Wakai, Akiko Tamakoshi

**Affiliations:** 1 Department of Hygiene, Fujita Health University School of Medicine, 1-98, Kutsukake-cho, Toyoake, Aichi 470-1192 Japan; 2 Department of Preventive Medical Sciences, Fujita Health University School of Health Sciences, Toyoake, Aichi Japan; 3 Department of Epidemiology for Community Health and Medicine, Graduate School of Medical Science, Kyoto Prefectural University of Medicine, Kyoto, Japan; 4 Public Health, Department of Social Medicine, Osaka University Graduate School of Medicine, Osaka, Japan; 5 Department of Environmental Epidemiology, Institute of Industrial Ecological Sciences, University of Occupational and Environmental Health, Kitakyushu, Japan; 6 Department of Preventive Medicine, Nagoya University Graduate School of Medicine, Nagoya, Japan; 7 Department of Public Health, Hokkaido University Graduate School of Medicine, Sapporo, Japan

**Keywords:** Biomarkers, Epidemiology

## Abstract

Primary prevention of premature death is a public health concern worldwide. Circulating microRNAs (miRNAs) have been described as potential diagnostic biomarkers for diseases as cancer and cardiovascular disease (CVD). This case-cohort study aimed to investigate the potential relationship between circulating miRNAs and the risk of premature death. A total of 39,242 subjects provided baseline serum samples in 1988–1990. Of these, 345 subjects who died of intrinsic disease (< 65 years old) and for which measurable samples were available were included in this study. We randomly selected a sub-cohort of 879 subjects. Circulatring miR-21, miR-29a, and miR-126 were determined using qRT-PCR. Conditional logistic regression models were used to analyse the data with respect to stratified miRNA levels. Multivariable logistic regression revealed that subjects with high circulating miR-21 and miR-29a individual levels had a significantly higher risk of total death, cancer death, and CVD death than those with medium miR-21 and miR-29a individual levels. Conversely, subjects with low circulating miR-126 levels had a significantly higher risk of total death than those with medium levels. This suggests that circulating miRNAs are associated with the risk of premature death from cancer and CVD, identifying them as potential biomarkers for early detection of high-risk individuals.

## Introduction

Premature death is broadly defined as the occurrence of mortality before the average age of death in a certain population. Although premature death (i.e. death before 65 years of age) accounts for only a small proportion of total mortality, its high impact on public health raises significant clinical and economic concerns. Despite the increasing average life expectancy in developed countries (e.g. currently over 80 years in Japan), a substantial proportion of adults still die prematurely^1–3^. Therefore, primary prevention of premature death is a considerable public health concern worldwide.

Over the past few decades, a considerable number of studies have been conducted on factors associated with premature death. Although the causes of these deaths vary, there appear to be some common causative factors^4,5^. For example, smoking cigarettes and/or being exposed to secondhand tobacco smoke are leading causes of premature death, and heavy drinking has also been implicated as a causative factor^6,7^. These lifestyles can increase the risk of cancer, heart disease, stroke, lung disease, and many other health problems that are associated with premature death. However, the identification of reliable biomarkers associated with premature death could complement the assessment of traditional risk factors and allow the identification of high-risk individuals with greater accuracy.

MicroRNAs (miRNAs), a class of small non-coding RNAs 18–25 nucleotides in length, are involved in a wide array of biological processes, including cell apoptosis, differentiation, development, proliferation, and metabolism. As miRNAs can be detected in bodily fluids (e.g., plasma, serum, urine, and saliva), there is much interest in studying the function and effects of these so-called “circulating miRNAs”. Several studies have demonstrated that circulating miRNAs can be used as biomarkers for various diseases such as cancers and cardiovascular disease (CVD)^8–14^. The circulating miRNAs miR-21, miR-29a, and miR-126 are particularly well studied due to their potential as novel biomarkers. For example, it has been reported that circulating level of miR-29a is correlated with clinical stage of colorectal cancer^15^. More recently, Yamada et al. reported that circulating miR-29a is upregulated in early stage of colorectal neoplasia, suggesting that circulating miR-29a may be increased in any clinical stages including early colorectal cancer^16^. miR-29a may be also useful for the prediction of type 2 diabetes^17^. Furthermore, Jiang et al. showed that miR-21 peripheral blood levels are increased in atherosclerosis, suggesting its potential as a biomarker for CVD^18^. In addition, miR-21 may be a potential biomarker for breast cancer and chronic kidney disease^10,19^. Also, Jianhong et al. showed that circulating miR-21 is up-regulated at all clinical stages in gastric cancer patients, suggesting that circulating miR-21 level is up-regulated throughout cancer progression^20^. Finally, circulating miR-126 has been reported as a potential biomarker for both type 2 diabetes^21^, non-alcoholic fatty liver disease^22^and lung cancer^23^ and may also reflect the progression of CVD^24^ As these diseases cause premature death, it is logical to hypothesise that circulating miR-21, miR-29a, and miR-126 levels may be associated with premature death.

We designed this study to investigate whether circulating miRNAs could represent new risk factors for premature death. The Japan Collaborative Cohort Study for Evaluation of Cancer Risk (JACC Study) is a large-scale population-based cohort study that has formed the basis of multiple reports on the relationships between disease risk and serum components, lifestyle, and living conditions of the Japanese population^25–28^. The JACC Study makes it possible to examine the significance of a predictive marker by analysing its levels in the serum of individuals who died prematurely due to various diseases such as cancer and CVD.

The present large prospective study examined the relationships between circulating miRNAs (miR-21, miR-29a, and miR-126) and premature death using a case-cohort study design as part of the JACC Study. Our results identify potential new risk factors for premature death that could complement the assessment of traditional risk factors and help to identify high-risk individuals with greater precision.

## Results

During the follow-up period, premature death was observed in 775 subjects, of which 345 had available baseline serum samples for analysis in this study. Of these premature deaths, 210 were due to cancer and 43 to coronary artery disease (CAD). Detailed data of the underlying causes of death as classified by the International Statistical Classification of Diseases and Related Health Problems 10th Revision (ICD-10) codes are described in Supplementary Tables S1-S3 online.

Table 1 shows the results of the statistical analysis comparing various characteristics of the premature death cases and controls. Perhaps unsurprisingly, the categories of age, sex, smoking status, and alcohol consumption displayed significant differences between the control and premature death groups (*p* < 0.001). Interestingly, serum levels of circulating miR-21 and miR-29a were significantly higher in the premature death group than in the control group (*p* = 0.001 and 0.01, respectively).

**Table 1:** Characteristics of subjects for the analysis of premature death.

	Control (n = 879)	Premature death (n = 345)	*p*
Age, years^a^	52.1 ± 7.3	49.5 ± 6.2	< 0.001
Female, n (%)	592 (67.3)	157 (45.5)	< 0.001
Body mass index, kg/m^2a^	23.1 ± 2.9	23.2 ± 3.2	0.65
Systolic blood pressure, mmHg^a^	128.2 + 15.7	129.0 ± 17.1	0.46
Educational level, years^a^	16.6 ± 2.0	16.7 ± 2.0	0.52
**Smoking status, n (%)**			
Never/ever	699 (79.5)	211 (61.2)	
Current	180 (20.5)	134 (38.8)	< 0.001
**Alcohol consumption, n (%)**			
Never/ever	517 (58.8)	155 (44.9)	
Current	362 (41.2)	190 (55.1)	< 0.001
Exercise, n (%)			
None	718 (81.7)	292 (84.6)	
> 1 h/wk	161 (18.3)	53 (15.4)	0.25
**Relative levels of circulating miRs**^**b**^			
Circulating miR-21	1.00 [0.46, 3.37]	1.39 [0.49, 5.78]	0.001
Circulating miR-29a	1.00 [0.46, 2.82]	1.41 [0.43, 5.65]	0.01
Circulating miR-126	1.00 [0.49, 2.30]	1.05 [0.44, 2.68]	0.63

^a^Data are expressed as mean value ± standard deviation.

^b^Data are expressed as relative values and 25th–75th percentiles in parentheses. The circulating miRNA levels were calibrated relative to controls.

Table 2 shows age- and sex-adjusted odds ratios (ORs) and 95% confidence intervals (CIs) for premature death with respect to the three categories of serum miRNA levels. Cases with high circulating miR-21 levels (> 75%) had a significantly higher risk of all death, cancer death, and CVD death than those with medium levels of this miRNA (OR [95% CI]: 1.93 [1.42–2.64], 1.74 [1.19–2.54], and 3.06 [1.42–6.74], respectively). Cases with high circulating miR-29a levels also had a significantly higher risk of all death, cancer death, and CVD death than those with medium levels of this miRNA (OR [95% CI]: 2.03 [1.48–2.78], 1.83 [1.25–2.69], and 3.13 [1.49–6.74], respectively). Interestingly, cases with low circulating miR-29a levels also had a significantly higher risk of all death and cancer death than those with medium levels of this miRNA (OR [95% CI]: 1.40 [1.01–1.94] and 1.56 [1.05–2.30], respectively). There were no significant associations with regard to circulating miR-126 levels. To control for the potential impact of subclinical symptoms of cancer at the time of baseline sampling, we repeated the analysis after excluding cases occurring within two years of the baseline survey. The results after this exclusion still showed a significant increase in the risk of premature death associated with serum levels of circulating miRNAs (data not shown).Table 3 shows the ORs and 95% CIs for premature death following analysis by the multivariable logistic regression model. Cases with high circulating miR-21 levels (> 75%) had a significantly higher risk of all death, cancer death, and CVD death than those with medium levels of this miRNA (OR [95% CI]: 1.98 [1.39–2.83], 1.84 [1.20–2.81], and 3.36 [1.39–8.33], respectively). Interestingly, cases with low circulating miR-21 levels also had a significantly higher risk of all death than those with medium levels of this miRNA (OR [95% CI]: 2.44 [1.02–5.94]). Cases with high circulating miR-29a levels had a significantly higher risk of all death, cancer death, and CVD death than those with medium levels of this miRNA (OR [95% CI]: 1.99 [1.39–2.84], 1.96 [1.27–3.00], and 3.23 [1.42–7.50], respectively). On the other hand, cases with low circulating miR-29a levels had a significantly higher risk of cancer death than those with medium levels of this miRNA (OR [95% CI]: 1.55 [1.03–2.34]). Cases with low levels of circulating miR-126 had a significantly higher risk of all death than those with medium levels of this miRNA (OR [95% CI]: 1.49 [1.06–2.10]). To control for the potential impact of subclinical symptoms of cancer at the time of baseline sampling, we repeated the analysis after excluding cases occurring within two years of the baseline survey. The results after this exclusion still showed a significant increase in the risk of premature death associated with serum levels of circulating miRNAs (data not shown).

**Table 2:** Age- and sex- adjusted ORs and 95% CIs for premature death with respect to the three categories of serum miRNAs levels.

	All death			Cancer death			CVD death		
	Case/control	OR (95% CI)	*p*	Case/control	OR (95% CI)	*p*	Case/control	OR (95% CI)	*p*
**miR-21**									
Low (< 25%)	71/212	1.02 (0.72–1.43)	0.92	42/212	1.00 (0.65–1.50)	0.98	12/212	1.92 (0.84–4.36)	0.12
Middle (25–75%)	136/422	Reference		85/422	Reference		13/422	Reference	
High (> 75%)	120/212	1.93 (1.42–2.64)	< 0.001	66/212	1.74 (1.19–2.54)	0.004	16/212	3.06 (1.42–6.74)	0.004
**miR-29a**									
Low (< 25%)	88/215	1.40 (1.01–1.94)	0.05	58/215	1.56 (1.05–2.30)	0.030	10/215	1.48 (0.62–3.46)	0.36
Middle (25–75%)	129/430	Reference		77/430	Reference		13/430	Reference	
High (> 75%)	118/215	2.03 (1.48–2.78)	< 0.001	65/215	1.83 (1.25–2.69)	0.002	18/215	3.13 (1.49–6.74)	0.003
**miR-126**									
Low (< 25%)	96/212	1.29 (0.94–1.77)	0.12	61/212	1.33 (0.91–1.93)	0.14	14/212	1.85 (0.86–3.97)	0.11
Middle (25–75%)	147/436	Reference		93/436	Reference		15/436	Reference	
High (> 75%)	96/219	1.37 (1.00–1.88)	0.05	47/219	1.05 (0.70–1.56)	0.81	14/219	2.04 (0.95–4.40)	0.07

Adjustment for sex, age, study site, systolic blood pressure, body mass index, smoking status, drinking status, leisure time activity, and educational level.

*CVD* cardiovascular disease.

**Table 3:** The ORs and 95% CIs for premarture death following analysis by the multivariable logistic regression model.

	All death			Cancer death			CVD death		
	Case/control	OR (95% CI)	*p*	Case/control	OR (95% CI)	*p*	Case/control	OR (95% CI)	*p*
**miR-21**									
Low (< 25%)	71/212	1.15 (0.80–1.65)	0.44	42/212	1.06 (0.68–1.64)	0.80	12/212	2.44 (1.02–5.94)	0.04
Middle (25–75%)	136/422	Reference		85/422	Reference		13/422	Reference	
High (> 75%)	120/212	1.98 (1.39–2.83)	< 0.001	66/212	1.84 (1.20–2.81)	0.01	16/212	3.36 (1.39–8.33)	0.01
**miR-29a**									
Low (< 25%)	88/215	1.40 (0.99–1.98)	0.06	58/215	1.55 (1.03–2.34)	0.04	10/215	1.40 (0.55–3.45)	0.46
Middle (25–75%)	129/430	Reference		77/430	Reference		13/430	Reference	
High (> 75%)	118/215	1.99 (1.39–2.84)	< 0.001	65/215	1.96 (1.27–3.00)	0.002	18/215	3.23 (1.42–7.50)	0.01
**miR-126**									
Low (< 25%)	96/212	1.49 (1.06–2.10)	0.02	61/212	1.49 (0.99–2.22)	0.05	14/212	2.14 (0.93–4.97)	0.07
Middle (25–75%)	147/436	Reference		93/436	Reference		15/436	Reference	
High (> 75%)	96/219	1.29 (0.90–1.86)	0.16	47/219	0.97 (0.61–1.52)	0.90	14/219	1.94 (0.81–4.60)	0.13

Adjustment for sex, age, study site, systolic blood pressure, body mass index, smoking status, drinking status, leisure time activity, and educational level.

*CVD* cardiovascular disease.

## Discussion

The present large prospective study examined associations between circulating miRNAs and premature death using a case-cohort study design. To our knowledge, this is the first study to show the association of circulating miRNA levels with premature death due to cancer and CVD. We observed that high serum levels of circulating miR-21 and miR-29a were most significantly associated with increased risk of premature death. On the other hand, low serum levels of circulating miR-126 were associated with increased risk of premature death. These results indicate that changes in serum levels of circulating miRNAs might be a novel risk factor preceding premature death events and could therefore be used as biomarkers to assess premature death risk many years before disease onset.

Over the past decades, many studies have examined environmental factors associated with the risk of premature death such as smoking and alcohol consumption^4,29–31^. We designed this prospective cohort study to identify new predictive factors independent of these lifestyle risk factors with the aim of improving prognostic information. A unique aspect of this study was the use of serum markers to identify factors associated with premature death. The focus on miRNA was particularly novel as these have not been previously assessed in this kind of research study. Although we adjusted our analysis for lifestyle risk factors such as smoking, exercise, and alcohol consumption, circulating miRNAs were significantly associated with premature death (Tables 2 and 3). These results suggest that circulating miRNA levels may represent novel factors for predicting premature death independently of traditional lifestyle-related risk factors.

The biological mechanisms of miR-21, miR-29a, and miR-126 have been described in multiple studies to date. MiR-21, one of the most widely studied abnormal miRNAs, is known to be upregulated in numerous tumours such as breast cancer, lung cancer, gastric cancer, colorectal cancer, hepatocellular carcinoma, pancreatic cancer, and ovarian carcinoma^32^. It has been proposed that circulating miRNAs such as miR-21 could be used as non-invasive biomarkers for cancer. Multiple studies have shown that the level of circulating miR-21 can distinguish cancer patients from healthy individuals and predict disease outcomes^33,34^. However, no previous studies have related miR-21 levels to the risk of developing cancer. Our findings showed that miR-21 serum levels were significantly associated with the risk of cancer death, suggesting that altered miR-21 levels may precede the onset of the disease. Indeed, many previous studies indicated that miR-21 plays an important role in the oncogenic process such as its association with inflammation, proliferation, apoptosis, invasion, and metastatic potential^32,35^. Also, these processes are well known to induce the development of metabolic dysfunction, an underlying cause of CVD^36^. The easily detectable imbalance of circulating miR-21 may therefore predict the pro-thrombotic, pro-inflammatory, pro-vasoconstrictive, and pro-proliferative phenotype that precedes the development of multiple clinical diseases, including cancer and CVD.

miR-29a has also been shown to regulate several biological functions underlying important physiological and pathological processes, including the cell cycle, proliferation, differentiation, apoptosis, and senescence^37^. A large body of literature has shown a significant role for miR-29a in various diseases, including cancer^38^. Although the majority of these studies have reported that miR-29a functions as a potent tumour suppressor, a few reports have demonstrated an oncogenic function of this miRNA. Circulating miR-29a has been reported as a promising biomarker for the early detection of cancer. However, higher levels of miR-29a in plasma conferred lower survival rates in lung cancer^39^. In addition, the pre-treatment level of circulating miR-29a is an independent prognostic marker for poor disease-free survival in hepatitis B virus-related HCC patients^40^. The evidence that miR-29a may have a dual role in regulating normal physiology and disease is consistent with the results of our study, which showed that both high and low levels of miR-29a are associated with premature death (Tables 2 and 3).

Our finding showed that low miR-126 levels correlated with lower premature death risk. This result may be explained by the fact that miR-126 prevents endothelial inflammation and dysfunction. Harris et al. indicated that miR-126 may control vascular inflammation by influencing leukocyte adhesion to endothelium via its effects on vascular cell adhesion molecule 1 expression^41^. Also, Jin et al. showed that circulating miR-126 levels were negatively correlated with levels of inflammatory cytokines such as tumour necrosis factor-α, interleukin (IL)-1β, and IL-6^42^. Another report showed that low plasma miR-126 levels might have a negative impact on vascular endothelial growth factor resistance and endothelial dysfunction in patients with type 2 diabetes^43^. Furthermore, increased levels of circulating miR-126 were reported to correlate with a lower predisposition to major adverse events in patients with stable CAD, a form of CVD^44^. Our finding showing that low miR-126 levels correlated with lower premature death risk is consistent with those of the previous reports because decreased levels of miR-126 may reflect endothelial dysfunction and subsequent impairment of the peripheral angiogenic system in the general population.

Although circulating miRNAs have been described as non-invasive biomarkers for multiple pathologies, including cancer and CVD, it is not clear whether the changes in miRNA levels precede the disease, appear at the early stages, or are a consequence of the disease state. Only a few studies have been performed based on large prospective cohorts in the general population, and little is known about the value of miRNAs as biomarkers for risk stratification of future events^45^. To our knowledge, the prognostic value of miRNAs for the general population has been evaluated in only two large-scale studies. Zampetaki et al. evaluated circulating miRNAs regarding their applicability in primary prevention of CVD. In 820 apparently healthy subjects, the three circulating miRNAs miR-126, miR-223, and miR-197 were associated with CAD in the general population, suggesting these miRNAs as biomarkers in primary prevention^46^. Also, Willeit et al. utilised the prospective population-based Bruneck Study (n = 810; survey year 1995) to show that circulating miR-122 is associated with the risk of developing metabolic syndrome and type 2 diabetes in the general population^47^.

In this study, we showed that circulating miRNAs (miR-21, miR-29a, and miR-126) are significantly associated with the risk of premature death due to cancer and CVD in the general population. To control for the potential impact of subclinical symptoms of cancer at the time of baseline sampling, we repeated the analysis after excluding cases occurring within two years of the baseline survey. Results after exclusion still showed a significant increase in the risk of premature death associated with serum levels of circulating miRNAs, suggesting that alterations in miRNA levels may precede the early stages of the disease. The findings of the current study combined with those of previous studies therefore suggest that changes in circulating miRNA patterns are not only the result of disease but may also precede the early stages of disease onset. Thus, circulating miRNAs may be useful tools as biomarkers for the risk of disease.

The cause and significance of changes in circulating miRNAs are important matters to be addressed in future studies. The risk of developing diseases such as cancer and CVD is known to result from the interaction between genetic and environmental factors. Given what is known about the action of miRNAs, it is easy to envisage how they could regulate gene expression related to environmental or lifestyle factors before, during, or after disease onset. For example, tobacco and alcohol consumption are leading risk factors in the development of diseases such as cancer, CVD, and liver injury. These lifestyles likely promote disease development by affecting gene expression and signalling pathways controlling important processes such as apoptosis, angiogenesis, and inflammation. These complex gene expression changes resulting from the interaction between genetic and environmental factors can be regulated by miRNAs. Thus, studying changes in circulating miRNAs may be crucial for our understanding of the interaction between genetic and environmental factors. In the future, it will be important to better elucidate the cause and significance of changes in circulating miRNAs, including the effect of genetic variations.

This study was only possible because of the exceptional characteristics of the JACC Study, a large-scale cohort study conducted in Japan. The following unique characteristics of the JACC Study allow us to research factors associated with premature death: (1) follow-up of the general population for approximately 20 years, (2) inclusion of relatively young individuals (64 years old or less) at baseline (about 72%), (3) availability of serum at baseline, i.e. before development of the fatal disease. Our results are valuable not only for their implications for premature death prediction but also for future research on novel mechanisms of premature death. However, there are some limitations to our study. First, since not all of the cohort participants provided blood samples, there was the possibility of selection bias. Although serum from 29,410 individuals was sampled at the age of 40–64 in the JACC Study, only 25,418 samples were currently available. Previously, we have used serum samples in case–control studies^48–50^. However, this study was conducted using serum samples that were not used in previous case–control studies. Indeed, 775 premature deaths were observed during the follow-up period, but baseline serum samples were only available for 345 of these subjects. Second, only Japanese subjects were recruited in this study, potentially biasing the results towards a particular ethnic group. The recruitment of other ethnic groups may result in different circulating miRNA profiles. Third, although we were able to show the link between circulating miRNAs and premature death from cancer and CVD, the sample size of this study did not allow us to perform analyses regarding particular cancer types and other causes of death (see Supplementary Table S2 online). Therefore, further detailed investigations are required to more thoroughly understand the association of circulating miRNAs with premature death.

In conclusion, this study successfully utilised a case-cohort study design to demonstrate for the first time that circulating miRNAs (miR-21, miR-29a, and miR-126) are associated with the risk of premature death from cancer and CVD. Our results have implications for early prediction of premature death as well as the advancement of research on its underlying mechanisms.

## Materials and methods

### Study subjects and data collection

Details of the concept and design of the JACC Study have been described elsewhere^27^. Briefly, the JACC Study was started between 1988 and 1990 and enrolled a total of 110,585 participants (46,395 men and 64,190 women) aged 40–79 years living in 45 areas across Japan. The participants responded to self-administered questionnaires regarding their lifestyle (such as smoking and alcohol consumption status) and medical history of major diseases (including liver disease, cancer, and CVD).

Peripheral blood samples were collected from a total of 39,242 participants during municipal health screening examinations. This study focused on the 29,410 individuals that were sampled at the age of 40–64, of which 25,418 serum samples were currently available for use, because we have used serum samples in case–control studies^48–50^.

Serum samples (taken at the baseline survey) were selected from 775 patients who died of intrinsic disease before the age of 65. Of these, 345 measurable samples were available for the present study. A sub-cohort was randomly selected from those aged 40–64 at the baseline sample measurement for which serum samples were available. A total of 926 subjects were selected as the sub-cohort (an extraction rate of 3.6%) by gender, age class (every 5 years), area, and sample collection year. Of these, 17 cases of premature death and 63 cases of death over 66 years old were included. Subsequently, subjects were excluded based on the following criteria: missing dates for analysis, technical issues with sample vials, and unconfirmed history of cancer and CVD. The final study population included 1,224 subjects (345 premature death cases and 875 control subjects). All participants provided written informed consent. The JACC Study began before the ethical guidelines were first established by the Japanese government in 2002, and the Japanese ethical guidelines allows such established epidemiological studies to continue without obtaining additional personal informed consent from kin or legal guardian^51–54^. The study design and use of serum were approved by the Ethical Board at Hokkaido University School of Medicine, where the central office of the JACC Study was located. This study was complied with guidelines of the Declaration of Helsinki.

### Follow-up

In the JACC Study, population registries in the municipalities were used for the follow-up of the subjects. In most areas, the follow-up was completed at the end of 2009; however, it was stopped at the end of 1999 in 4 areas, at the end of 2003 in another 4 areas, and at the end of 2008 in 2 areas. We discontinued the follow-up of those who had moved out of their area after the baseline survey. Individuals who re-located away from the study area were treated as dropouts, as deaths could not be confirmed in our follow-up system. We reviewed death certificates and classified the underlying causes of death as coded by the National Vital Statistics according to the ICD-10 (http://www.who.int/classifications/icd/en/). Cancer was defined using codes C00-C97, and CVD using codes I00-I99. All deaths that occurred in the cohort were ascertained by death certificates from public health centres.

### Measurement of circulating miRNAs

Serum was stored at − 80 °C, as this method has been proven to reliably maintain circulating miRNA levels^55^. Serum levels of the three circulating miRNAs were detected by quantitative real-time polymerase chain reaction (qRT-PCR) following a procedure that has been described in detail elsewhere^56^ In brief, serum miRNAs were isolated using TRIzol reagents according to the manufacturer’s protocol (Invitrogen, Foster City, USA). qRT-PCR was then performed with specific primers for miRNAs in an ABI PRISM 7900 Sequence Detection System (Applied Biosystems, Foster City, CA, USA) using a miScript System (Qiagen, Valencia, CA, USA) according to the manufacturer's instructions. Relative expression levels of miRNAs were calculated using the comparative cycle threshold method. *Caenorhabditis elegans* miR-39 (cel-mir-39) was used to spike RNA samples as an external control to check the efficiency of either the RNA extraction or the cDNA synthesis. This is a widely used practical method in the measurement of circulating miRNAs^57,58^.

### Analysis

Statistical analyses were based on mortality during the follow-up period (1989–2009). To compare the baseline characteristics of premature death cases and control subjects, the paired *t*-test was used to test mean values, and the McNemar test was used for percentages of premature death risk factors. Group circulating miRNA levels were compared by Wilcoxon tests.

ORs for premature death were estimated using conditional logistic regression models. Subjects were categorised into 3 groups according to their serum circulating miRNA levels. The categories used in analyses of the distribution of sub-cohort subjects were defined as: < 25% (low), 25–75% (medium), and > 75% (high). miRNA levels in the premature death group were then categorised as low, medium, or high if they were < 25%, 25–75%, or > 75% of the average level of the sub-cohort (control) group, respectively. Multivariable logistic regression modelling was then performed to estimate the ORs and 95% CIs for premature death using the medium group (25–75%) as a reference group. Covariates for adjustment included sex, age, area of residence, body mass index (categorised as < 18.5 kg/m^2^, 18.5–24.9 kg/m^2^, and ≥ 25 kg/m^2^), systolic blood pressure (mm Hg), cigarette smoking status (never, former, and current), alcohol consumption status (never, ex-, and current), walking (≥ 30 min/day or not), exercise (≥ 1 h/week or not), and educational level (attended school up to 15–18 years old or > 18 years old). Data for the above factors were self-reported. For all covariates, missing values were included in the model as an additional category of variable. The data were analysed with R version 3.5.1 statistical software (R Core Team (2019). R: A language and environment for statistical computing. R Foundation for statistical Comuting, Vienna, Austria. https://www.R-project.org/)^59^. All statistical tests were 2-tailed, and a *p*-value of less than 0.05 was considered statistically significant.

## Supplementary Information

Supplementary Information.
