# Supplementary material for: Circulating miR-21, miR-29a, and miR-126 are associated with premature death risk due to cancer and cardiovascular disease: the JACC Study
Source: Sci Rep. 2021 Mar 5;11:5298. doi: 10.1038/s41598-021-84707-7 (PMC7935984; doi:10.1038/s41598-021-84707-7)
Supplement: Supplementary file 1 — Supplementary Information. [file 41598_2021_84707_MOESM1_ESM.docx]

**Supplementary Information**

**Circulating miR-21, miR-29a, and miR-126 are associated with premature death risk due to cancer and cardiovascular disease: the JACC Study**

Hiroya Yamada^1*^, Koji Suzuki^2^, Ryosuke Fujii^2^, Miyuki Kawado^1^, Shuji Hashimoto^1^, Yoshiyuki Watanabe^3^, Hiroyasu Iso^4^, Yoshihisa Fujino^5^, Kenji Wakai^6^, Akiko Tamakoshi^7^ & the JACC Study Group

Supplementary Table S1. Premature death subjects and classified the underlying causes of death coded for ICD10.

| ICD-10 Code | Premature death (n=345) | |
| --- | --- | --- |
|  | n | % |
| A | 1 | 0.29 |
| B | 4 | 1.16 |
| C | 206 | 59.71 |
| D | 10 | 2.90 |
| E | 4 | 1.16 |
| F | 1 | 0.29 |
| G | 12 | 3.48 |
| H | 0 | 0.00 |
| I | 43 | 12.46 |
| J | 22 | 6.38 |
| K | 16 | 4.64 |
| L | 0 | 0.00 |
| M | 7 | 2.03 |
| N | 2 | 0.58 |
| Q | 1 | 0.29 |
| R | 3 | 0.87 |
| S | 2 | 0.58 |
| T | 7 | 2.03 |

Supplementary Table S2. Cancer death subjects and classified the underlying causes of death coded for ICD10

| ICD-10 Code | Cancer (n = 206) | n | % |
| --- | --- | --- | --- |
|  |  |  |  |
| C00-14 | Malignant neoplasms of lip, oral cavity and pharynx | 6 | 2.9 |
| C15-26 | Malignant neoplasms of digestive organs | 102 | 49.5 |
| C30-39 | Malignant neoplasms of respiratory and intrathoracic organs | 30 | 14.6 |
| C40-41 | Malignant neoplasms of bone and articular cartilage | 0 | 0.0 |
| C43-44 | Melanoma and other malignant neoplasms of skin | 1 | 0.5 |
| C45-49 | Malignant neoplasms of mesothelial and soft tissue | 3 | 1.5 |
| C50 | Malignant neoplasm of breast | 15 | 7.3 |
| C51-58 | Malignant neoplasms of female genital organs | 10 | 4.9 |
| C60-63 | Malignant neoplasms of male genital organs | 0 | 0.0 |
| C64-68 | Malignant neoplasms of urinary tract | 3 | 1.5 |
| C69-72 | Malignant neoplasms of eye, brain and other parts of central nervous system | 3 | 1.5 |
| C73-75 | Malignant neoplasms of thyroid and other endocrine glands | 1 | 0.5 |
| C76-80 | Malignant neoplasms of ill-defined, secondary and unspecified sites | 4 | 1.9 |
| C81-96 | Malignant neoplasms, stated or presumed to be primary, of lymphoid, haematopoietic and related tissue | 28 | 13.6 |
| C97 | Malignant neoplasms of independent (primary) multiple sites | 0 | 0.0 |

Supplementary Table S3. CVD death subjects and classified the underlying causes of death coded for ICD10

| ICD-10 Code | CVD (n = 43) | n | % |
| --- | --- | --- | --- |
|  |  |  |  |
| I00-02 | Acute rheumatic fever | 0 | 0.0 |
| I05-09 | Chronic rheumatic heart diseases | 0 | 0.0 |
| I10-15 | Hypertensive diseases | 1 | 2.3 |
| I20-25 | Ischaemic heart diseases | 9 | 20.9 |
| I26-28 | Pulmonary heart disease and diseases of pulmonary circulation | 1 | 2.3 |
| I30-52 | Other forms of heart disease | 10 | 23.3 |
| I60-69 | Cerebrovascular diseases | 20 | 46.5 |
| I70-79 | Diseases of arteries, arterioles and capillaries | 2 | 4.7 |
| I80-89 | Diseases of veins, lymphatic vessels and lymph nodes, not elsewhere classified | 0 | 0.0 |
| I95-99 | Other and unspecified disorders of the circulatory system | 0 | 0.0 |
